# Effect of human hydrosalpinx fluid in fertilization rate of mouse oocyte and embryo quality

**DOI:** 10.5935/1518-0557.20210118

**Published:** 2023

**Authors:** Yada Thongyou, Pareeya Somsak, Waraporn Piromlertamorn, Usanee Sanmee

**Affiliations:** 1 Division of Reproductive Medicine, Department of Obstetrics and Gynecology, Faculty of Medicine, Chiang Mai University, Chiang Mai 50200, Thailand; 2 CMEx fertility center, Center of Medical Excellence, Chiang Mai University, Chiang Mai 50200, Thailand

**Keywords:** hydrosalpinx fluid, oocyte, fertilization rate, embryo quality

## Abstract

**Objective:**

To determine whether human hydrosalpinx fluid might have a deleterious effect on the fertilization rate and embryonic development of the exposed mouse oocytes.

**Methods:**

Mouse cumulus-oocyte complexes (COCs) were randomly allocated for exposure to pure hydrosalpinx fluid (100% HSF group, n=400), EBSS containing 50% of hydrosalpinx fluid (50% HSF group, n=320) and pure EBSS (control group, n=300).

**Results:**

The results showed that the fertilization rate in the 100% HSF group was significantly lower than the control group (64.0% versus 73.0%, *p*=0.031). The blastocyst formation rate was also lower in the 100% HSF group than 50% HSF and the control group (51.5% versus 56.9% versus 56.3%, respectively), but not statistically significant (*p*=0.275). There was no significant difference in the mean numbers of cells in the ICM, TE, and total cell number in blastocysts from the control group and two hydrosalpinx fluid exposure groups.

**Conclusions:**

Human hydrosalpinx fluid has a negative effect on the fertilization rate of the exposed mouse oocytes. However, this effect was found only in undiluted concentration and does not affect the subsequence of embryonic development and blastocyst cell number.

## INTRODUCTION

In addition to infertility, the presence of hydrosalpinx has adverse effects on assisted reproductive technology (ART) outcomes (Zeyneloglu *et al*., 1998; Camus *et al*., 1999). Several mechanisms have been proposed include a direct embryotoxic effect, alteration endometrial receptivity, and mechanical flushing of the embryo from hydrosalpinx fluid leakage into the uterine cavity. Therefore, treatment of hydrosalpinx by either laparoscopic salpingectomy or proximal tubal occlusion before in vitro fertilization (IVF) increases the pregnancy rates (D’Arpe *et al*., 2015; Tsiami *et al*., 2016). Concerning the effect of salpingectomy on the ovarian blood flow (Grynnerup *et al*., 2013) and subsequent reduced ovarian response, proximal tubal occlusion seems to be an interesting alternative to salpingectomy. In case of inability to perform surgery or develop hydrosalpinx during controlled ovarian stimulation, ultrasound-guided aspiration of hydrosalpinx at oocyte collection is an option (Zhou *et al*., 2016).

The drawbacks of having the dilated tube during the IVF process interferes with the accessibility of the ovary during oocyte retrieval. Furthermore, accidental exposure of oocytes to hydrosalpinx fluid can happen during the procedure. Our PubMed search revealed only one study on the effect of hydrosalpinx fluid on oocytes and their only focus on the outcome of fertilization rates (de Vantéry Arrighi *et al*., 2001). This study aimed to determine whether human hydrosalpinx fluid might have a deleterious effect on oocytes, fertilization and subsequent early embryonic development using a mouse model.

## MATERIAL AND METHODS

### Hydrosalpinx fluid

Hydrosalpinx fluid (HSF) was collected in an aseptic manner from hydrosalpinx of six patients undergoing laparoscopic surgery for infertility. After the presence of hydrosalpinx was confirmed laparoscopically, a sterile needle was used to aspirate hydrosalpinx fluid through the fallopian tube lumen. The hydrosalpinx fluid specimen was immediately stored at-70°C until used. Before use, hydrosalpinx fluid was warmed to 37°C, and then 2 ml of hydrosalpinx fluid was used for routine bacterial culture, pH and osmolarity check. The pH valued was determined by a pH meter (GonotecOsmomat 030; Gonotec GmbH, Berlin, Germany). The osmolarity was determined by a digital osmometer (Starter 3100 pH Bench; Ohaus Corporation, New Jersey, USA). The remaining hydrosalpinx fluid was used in the experiment. Mouse embryo assay was repeated six times, each time using hydrosalpinx sample from one patient.

### Experimental animals

Outbreed female and male International Cancer Research (ICR) mice were obtained from the National Animal Institute, Mahidol University, Bangkok, Thailand. They were cared for at Animal Husbandry Unit, Faculty of Medicine, Chiang Mai University. All procedures related to mice followed the international and national guidelines for ethical conduct in the care and use of animals for research. The room was adequate ventilation at 25±2°C, under humidity of 60-70%, and controlled 12-hour light/12-hour dark cycles. The mice were rested, not disturbed for seven days before the experiment to avoid the effect of stress from transportation. The Animal Ethics Committees of the Faculty of Medicine, Chiang Mai University approved the use of mice in our study under approval no. 28/2563.

### Collection of cumulus-oocyte complexes (COCs) and exposure with hydrosalpinx fluid

Five- to nine-week-old female mice were super-ovulated by an intraperitoneal (IP) injection of 10 IU pregnant mare serum gonadotropin (PMSG; Sigma, St. Louis, MO, USA), followed 48 hours later by an IP injection of 10 IU human chorionic gonadotropin (Pregnyl, Organon, Oss, The Netherlands). Sixteen hours after the second injection, the mice were killed by cervical vertebrae dislocation. The peritoneal cavity was exposed, and the two oviducts were aseptically removed and placed in Earle’s Balanced Salts Solution (EBSS; Biological Industries, Kibbutz Beit Haemek, Israel), containing 0.5% bovine serum albumin (BSA; Sigma, St Louis, MO). Cumulus-oocyte complexes (COCs) were removed from the oviduct and separated into three groups for the experiment.

To assess the effect of hydrosalpinx fluid on oocytes, two experimental groups and a control group were studied;100% HSF group: pure hydrosalpinx fluid, 50% HSF group: EBSS containing 50% of hydrosalpinx fluid, and control group: pure EBSS. The COCs were exposed to the assigned condition for five minutes. Following the exposure, COCs were washed in EBSS and transferred to 50 µl drops of fertilization medium (Cook, Brisbane, Australia) under mineral oil (IrvineScientific, USA) and use for IVF. All steps were done in an IVF chamber (HD Scientific, NSW, Australia) under an atmosphere of 6% CO_2_ at 37°C.

### *In vitro* fertilization and embryo culture

Male ICR mice aged 10-12 weeks were killed by cervical vertebrae dislocation. Both cauda epididymis was removed and placed in 1 ml of fertilization medium (Cook). Capacitation was allowed to proceed for 30 minutes in an atmosphere of 6% CO_2_, 5% O_2_, and 89% N_2_ at 37°C. The spermatozoa were transferred to COCs drops for insemination at a final motile sperm concentration of 2.5x10^5^ /ml. All experiment groups from the same hydrosalpinx sample were used spermatozoa obtained from the same male. Two hours later, MII oocytes were transferred to culture in 10 µl drop of cleavage medium (G1-plus; Vitrolife, Sydney, Australia) under mineral oil (IrvineScientific). The fertilization rate was determined the next day by counting the number of two-cell embryos. Seventy-two hours post insemination, the embryos were transferred to blastocyst medium (G2-plus; Vitrolife, Sydney, Australia) under mineral oil (Irvine Scientific) and cultured in similar conditions for 48 hours. Embryo development was evaluated under an inverted microscope every 24 hours until completion of 120 hours. Mouse blastocysts were classified as early, partial, full, expanding, hatching and hatched blastocysts, using the criteria proposed by Gardner *et al*. (2000) for human blastocyst development.

### Differential staining of the inner cell mass (ICM) and trophectoderm cell (TE)

Differential staining was performed on all expanding, hatching, and hatched blastocysts, using the protocol described by Pampfer *et al*. (1990). In brief, the blastocyst with intact zona was placed in a 0.5% pronase for ten minutes to remove zona pellucida. The zona-free blastocysts were washed three times in calcium- and magnesium-free buffer, before exposure to rabbit anti-mouse antibody (Sigma M5774; concentration 1:50) for 30 minutes at 37°C. Then washed and transferred into guinea pig complement serum (Sigma S1639) with propidium iodide (Sigma P4170) and bisbenzimide (Sigma B2261) at 37°C for 10-15 minutes. The blastocysts were washed and transferred onto glass slides to allow air drying. The slides were mounted in glycerol, and the numbers of the ICM and the TE were counted using a Nikon E600 epifluorescence microscope, equipped with the LUCIA FISH program (Laboratory Imaging, Prague, Czech Republic). The ICM nuclei were observed to stain blue while the TE nuclei showed intense pink color.

### Statistical analysis

Statistical analysis was performed using SPSS program version 16. The Chi-square test in R × C list data was used to compare fertilization rate and blastocyst formation rate in three groups. The mean numbers of ICM and TE cells were compared by one-way analysis of variance (ANOVA) when data distribution was normal or the Kruskal Wallis test when normality could not be confirmed. A two-tailed *p*<0.05 was considered statistically significant.

## RESULTS

One thousand and twenty COCs were included in the study. Of these, 400 COCs were exposed to 100% HSF, 320 COCs were exposed to 50% HSF, and 300 COCs were exposed to EBSS as the control. A significantly lower fertilization rate was observed in the 100% HSF group compared to the control group (64.0% *versus* 73.0%, *p*=0.031, Table 1). The fertilization rate was comparable in the 50% HSF group compared to the control group (68.1% *versus* 73.0%, *p*=0.394). The blastocyst formation rate was lower in the 100% HSF group than the 50% HSF and the control group (51.5% *versus* 56.9% *versus* 56.3%, respectively), but not statistically significant (*p*=0.275, Table 1). The numbers of embryos in various stages of blastocyst development after 120 hours of culture are shown in Table 2.

**Table 1:** Fertilization and blastocyst formation rate for COCs exposed to control, 50% and 100% hydrosalpinx fluid.

Variable	Control	50% HSF	100% HSF	p
Number of COCs	300	320	400	-
Fertilization rate	219 (73.0) *	218 (68.1)	256 (64.0) *	0.041
Blastocyst formation rate	169 (56.3)	182 (56.9)	206 (51.5)	0.275

Values are presented as number (%)

* Chi-square test, *p*=0.031

**Table 2:** Mouse embryo development after 120 hours of culture.

Variable	Control (n=300)	50% HSF (n=320)	100% HSF (n=400)	*p* *
Early blastocyst	25 (8.3)	14 (4.4)	20 (5.0)	0.085
Partial blastocyst	57 (19.0)	55 (17.2)	57 (14.3)	0.446
Full blastocyst	0	0	0	-
Expanding blastocyst	4 (1.3)	5 (1.6)	4 (1.0)	0.871
Hatching blastocyst	61 (20.3)	80 (25.0)	93 (23.3)	0.170
Hatched blastocyst	22 (7.3)	28 (8.8)	32 (8.0)	0.755

Values are presented as number (%)

* Chi-square test

There was no significant difference in the mean numbers of cells in the ICM (Kruskal Wallis test, *p*=0.415), TE (ANOVA, *p*=0.945), and total cell number (Kruskal Wallis test, *p*=0.363) in blastocysts from the control group and two HSF exposure groups (Table 3). Likewise, the ICM to TE ratio was not different among the three groups (Kruskal Wallis test, *p*=0.479).

**Table 3:** Numbers of cells in the ICM, TE, total cell number, and the ICM to TE ratio in control, 50%, and 100% hydrosalpinx fluid exposure groups.

Variable	Control	50% HSF	100% HSF	p
ICM *	18.44±4.06	18.57±4.81	19.40±4.99	0.415
TE^†^	72.13±17.76	71.40±18.53	72.41±16.50	0.945
Total cells *	90.57±18.11	89.97±19.97	91.80±18.57	0.363
ICM/TE *	0.28±0.19	0.27±0.10	0.28±0.08	0.479

Values are presented as mean ± standard deviation

* Kruskal Wallis test

† ANOVA test

Hydrosalpinx fluid was obtained from six infertile patients. The characteristic of each sample in pure and after dilution to 50% with EBSS is shown in Table 4. The color is varying from clear to red or brown. The pH valued of pure hydrosalpinx fluid ranged from 7.39-7.43 (within physiologic range) in sample no.4-6 to 7.68-7.85 (alkaline) in sample no.1-3. The osmolarity of pure hydrosalpinx fluid is varied from a physiologic range of 275-278 mOsm in samples no.2 and 5 to a hypoosmotic range of 269-270 mOsm in samples no.1 and 6, and hyperosmotic range of 309-330 mOsm in samples no.3 and 4. Routine bacterial culture of all hydrosalpinx fluid specimens showed no bacterial growth.

**Table 4:** Characteristics of hydrosalpinx fluid from six infertile patients.

Sample number	Color	pH		Osmolarity (mOsm/kg)		Culture
		100% HSF	50% HSF	100% HSF	50% HSF	
1	light red	7.68	7.52	270	281	no growth
2	brown	7.85	7.69	275	292	no growth
3	clear	7.84	7.73	330	311	no growth
4	brown	7.39	7.49	309	293	no growth
5	clear	7.39	7.35	278	265	no growth
6	clear	7.43	7.42	269	268	no growth

**Figure 1:**
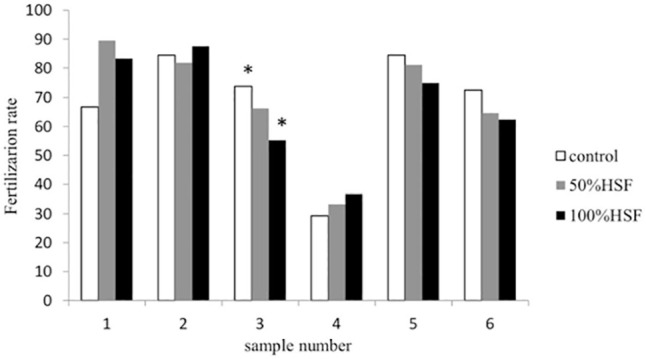
Fertilization rate for COCs exposed to control, 50% and 100% hydrosalpinx fluid categorized by sample. * Chi-square test, *p*=0.042.

**Figure 2:**
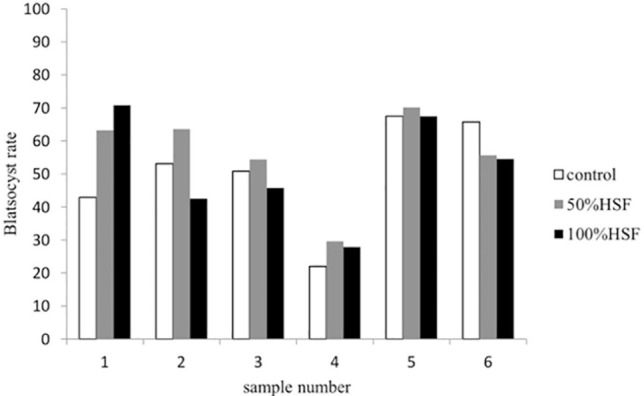
Blastocyst formation rate for COCs exposed to control, 50% and 100% hydrosalpinx fluid categorized by sample. Not statistically significant.

When categorized by hydrosalpinx fluid sample, the COCs exposed to the 100% HSF sample no.3 significantly decreased the fertilization rate compared to the control (55.2 *versus* 73.9%, *p*=0.042), whereas the COCs exposed to other hydrosalpinx fluid samples did not affect the fertilization rate (Figure 1). The blastocyst formation rate was not significantly different between COCs exposed to hydrosalpinx fluid no.1-6 in both 50% and 100% concentration compared with their respective controls (Figure 2).

## DISCUSSION

This study was designed to use the mouse model to assess the influence of human hydrosalpinx fluid on the ability of oocytes to fertilized and subsequence embryonic development. We designed to exposed oocytes to hydrosalpinx fluid for five minutes. This methodology mimics the real condition in clinical practice that accidental exposure of oocytes to hydrosalpinx fluid during oocyte retrieval procedure was not longer than five minutes. Likewise, in terms of the hydrosalpinx fluid concentration, we designed to expose the oocytes with both pure hydrosalpinx fluid to see the real effect, and 50% diluted hydrosalpinx fluid mimics the real condition in which the follicular fluid from the retrieved oocyte will dilute the effect of the hydrosalpinx fluid. The current evidence supports that hydrosalpinx reduces the IVF success rate, but the exact mechanism remains unclear. One proposes mechanism is a direct cytotoxic effect on embryos from the composition of inflammatory substances, microorganisms and free radicals (Chen *et al*., 2002; Beyler *et al*., 1997; Rawe *et al*., 1997). This means that it inevitably affects the oocytes as well. To our knowledge, only one study has been performed regarding the effect of hydrosalpinx fluid on oocytes. de Vantéry Arrighi *et al*. (2001) pre-incubated the COCs with media containing 50% HSF for one hour before IVF and focus on the fertilization rate. They found that exposure to 50% HSF did not affect the oocytes fertilization rate in all three hydrosalpinx fluid samples, the mean fertilization rate was 71.5% compared to 73.1% of nonexposed groups. Our study also found the same that oocytes exposed to 50% HSF have a fertilization rate comparable to oocytes that are not exposed to hydrosalpinx fluid (68.1% *versus* 73.0%, *p*=0.394). Furthermore, we continued to observe and found that the blastocyst formation rates (56.9% *versus* 56.3%, *p*=0.974) and their cell number are also comparable. This the first reported study on the effect of pure HSF on the exposed oocytes. The results show a significantly lower fertilization rate of oocytes exposed to pure HSF for five minutes before IVF compared to the control (64.0% *versus* 73.0%, *p*=0.031), and found a tendency of lower blastocyst formation rate (51.5% *versus* 56.3%, *p*=0.275) although not statistically significant. Therefore, hydrosalpinx fluid may have a direct cytotoxic effect on oocytes, and this effect can be obscured by the effect of the dilution.

The mechanisms for explaining the toxicity of hydrosalpinx fluid on oocytes remain questionable. The deviated pH and osmolarity of hydrosalpinx fluid from the physiologic range is one of the potential mechanisms. Stability in optimal pH and osmolarity is a vital factor for cell homeostasis and intracellular process including protein synthesis, mitochondrial function, and cytoskeletal regulation. Nevertheless, metaphase II oocytes strongly regulate alkalinity but are unable to compensate for acidosis. The intracellular pH of the oocyte rapidly recovered to a physiologic pH after exposure to the external pH environment up to pH 8.0 without any negative effects (Dale *et al*., 1998). Fortunately, most of the hydrosalpinx fluid is weakly alkaline, therefore, the high pH value should not be the primary mechanism of the adverse effect of hydrosalpinx fluid on oocytes. Also, it has been reported that correction of pH of hydrosalpinx fluid to the physiologic range before exposed to mouse embryos did not improve blastocyst rates (Mukherjee *et al*., 1996). Exposing oocytes to anisosmotic condition induced cell volume changes and destruction of the metaphase II spindles. However, Van den Abbeel *et al*. (2007) found that after exposure of human metaphase II oocytes to solutions in an osmolarity range between 39 and 2,264 mOsm for five minutes, there was no deleterious effect on the fertilization rate and further embryonic development. Most hydrosalpinx fluids demonstrated osmolarity within the physiologic range. Although some hydrosalpinx fluid is not, but only a little deviation. Therefore, osmolarity change in hydrosalpinx fluid does not explain the negative effect on oocytes.

Another possible mechanism is 1) impaired the essential nutrients for the cytoplasmic maturation of the oocytes. Hydrosalpinx fluid contains a composition similar to those in serum but the steroid hormones, glucose, and proteins that necessary for development are lower than follicular fluid (Mukherjee *et al*., 1996), 2) increasing in the various inflammatory cytokines (Strandell *et al*., 2004) in response to a long-term infection and inflammation of the fallopian tube may detriment to the oocytes, and 3) endotoxin produced by the microorganism may direct action on oocytes resulting in the disruption on the intracellular metabolic pathway (Bidne *et al*., 2018) lead to the impair of oocyte maturation which is essential for successful fertilization and embryonic development. To confirm all these possible mechanisms, further in-depth analysis of the hydrosalpinx fluid is necessary.

Further analysis on the effect of hydrosalpinx fluid on each oocyte was conducted independently. We found that only one out of six human hydrosalpinx fluid samples have a deleterious effect on the fertilization rate of the exposed mouse oocytes, while the other five samples were not affected. Human hydrosalpinx fluid comprises a heterogenous nature with an individual variation in the components such as electrolytes, cytokines, nutrients, as well as endotoxin (Bao *et al*., 2017). The adverse effects can be attributed to an abnormality in the hydrosalpinx fluid components themselves, not all hydrosalpinx fluid. Therefore, hydrosalpinx fluid from different patients produce different effects on the exposed oocytes. Nevertheless, at least some hydrosalpinx fluid can harm oocytes. From the current knowledge, no indicators can predict the negative effect of hydrosalpinx fluid, whether in the gross appearance, color, or even a biochemical analysis of its components. Moreover, no common toxic factor or pathologic microorganisms have been detected in previously published studies. Therefore, universally avoiding exposure to all hydrosalpinx during oocyte retrieval procedure is the best option.

In conclusion, human hydrosalpinx fluid has a negative effect on the fertilization rate of the exposed mouse oocytes. However, this effect was found only in undiluted concentration and does not affect the subsequent embryonic development and blastocyst cell number.
